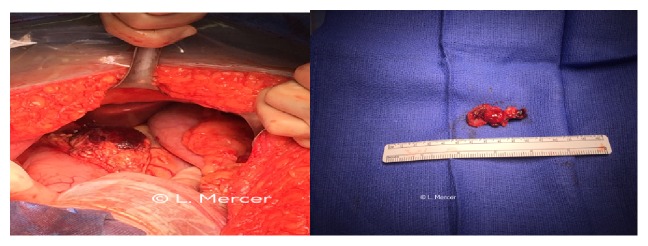# Splenic Artery Aneurysm Case Report

**DOI:** 10.1155/2019/8347983

**Published:** 2019-03-19

**Authors:** Pouya Abhari, Sina Abhari, Anwar Jackson, Ahmed S. Z. Moustafa, Leo Mercer, Mohammad Ashraf

**Affiliations:** ^1^Hurley Medical Center, Flint, Michigan, USA; ^2^Michigan State University College of Human Medicine, East Lansing, Michigan, USA

## Abstract

Splenic artery aneurysm rupture is a rare complication of pregnancy with very high maternal and fetal mortality rate. In this paper, a case of splenic artery aneurysm rupture at 34 weeks of gestation with both maternal and fetal survival is presented.

## 1. Introduction

Splenic artery aneurysms are the third most common abdominal aneurysms, behind infrarenal aorta and iliac artery aneurysms; however the actual incidence of the disease ranges from 0.01% to 0.98 [1–3]. Splenic artery aneurysm rupture is an even rarer event occurring in only 2 to 3% of all splenic artery aneurysms [1, 2]. Despite the low overall rates of splenic artery aneurysm rupture, some estimate that 20 to 40% of such cases occur in pregnant women who otherwise have no other comorbidities [2]. Hormonal changes in pregnancy have been speculated as a potential reason for the increased risk of splenic artery aneurysm, with estrogen, progesterone, and relaxin weakening arterial walls. Rupture of splenic artery aneurysm in pregnant women has significant maternal and fetal mortality, with mortality rates of 75% and 95%, respectively [2, 4]. The rupture can occur as a sudden single stage or in two stages, with the later resulting from the initial rupture being contained in the lesser sac before it spreads into the greater sac [5]. Two-stage rupture of splenic artery aneurysm occurs in 20 to 25% of all cases of splenic artery aneurysm rupture and usually presents as sudden abdominal pain followed by a period of clinical stability followed by sudden collapse. This article describes such a case. In this article, we will discuss a report of splenic artery aneurysm rupture in a patient at 34 weeks of gestation with good maternal and fetal outcomes.

## 2. Case Report

The patient is a 34-year-old Caucasian female at 34 weeks and 1 day of gestation who presented to triage with complaint of abdominal pain. The pain was acute with onset two hours prior to triage visit. Pain was mainly localized to epigastrium and left upper quadrant associated with nausea, exacerbated by movement.

Physical exam revealed abdominal tenderness mainly in the epigastric area without peritoneal signs of rebound or guarding. Abdomen was also distended. The patient suddenly became lethargic, hypotensive, and tachycardic; fetal heart tones revealed heart rate in 40s range.

An emergent exploratory laparotomy with cesarean delivery under general anesthesia was performed for indication of nonreassuring fetal heart tones and suspected hemorrhagic shock. The massive transfusion protocol was activated as per hospital protocol, and the acute care surgery was immediately consulted. Upon entering the peritoneal cavity 800 cc of blood was encountered. The fetus was delivered via low transverse hysterotomy, the abdomen was then packed. Upon further exploration of the abdomen by surgical team, splenic artery aneurysm rupture was diagnosed (Figure 1) and patient underwent a splenectomy and partial pancreatectomy. The patient was admitted to ICU post-op and was discharged in stable condition on postoperative day 6. The fetus was admitted to NICU secondary to respiratory distress and low Apgar scores and was discharged home on 10 days of age.

## 3. Discussion

The extremely high maternal and fetal mortality associated with splenic artery aneurysm rupture highlight the importance of understanding the pathophysiology. Unfortunately, a complete understanding of the splenic artery aneurysm disease process is impeded by the rarity of the condition [6]. Possible risk factors for splenic artery aneurysm include portal hypertension, previous liver transplantation, pancreatitis, and pregnancy [6]. Physiological changes in pregnancy that are believed to increase the risk of splenic artery aneurysm include increased portal venous pressure, increased cardiac output, and increased hormone levels that weaken the arterial wall structure [2]. Once splenic artery aneurysms develop, rapid growth of the aneurysm or diameter greater than 2 cm both increase the risks of rupture [6].

Detection of splenic artery aneurysms is usually incidental, and the rarity of the event precludes any recommendations for routine screening, even in pregnancy [2, 6]. Most splenic artery aneurysms are asymptomatic, while others usually present with vague symptoms such as nausea, vomiting, and abdominal pain [2]. When splenic artery aneurysms are suspected, angiography is the gold standard for diagnosis [4]. CT and MRI scans are useful for 3D evaluation of aneurysms, and even X-ray can detect splenic artery aneurysms with calcifications [5]. In pregnancy, however, ultrasound is the preferred diagnostic modality. Ruptured splenic artery aneurysms present with hypotension, loss of consciousness, and other signs of hemorrhagic shock [5]. Due to the rarity of the condition, splenic artery aneurysms ruptures are often misdiagnosed as uterine rupture and placental abruption in pregnant patients, with the correct diagnosis being delayed until the time of laparotomy [5].

Management of splenic artery aneurysms depends on their size, location, and presenting symptoms. In nonpregnant patients, splenic artery aneurysms larger than 2 cm, regardless of symptoms, should be treated. Techniques for treating splenic artery aneurysms in nonpregnant patients include laparoscopic ligation or resection, transcatheter embolization, and percutaneous angiographic embolization [1]. In pregnant patients, splenic artery aneurysms of any size should be treated operatively [1]. Minimally invasive operative techniques such as operative occlusion, resection, and arterial bypass can be safely performed in stable pregnant patients [1]. The mortality rate of nonemergent treatment of splenic artery aneurysms ranges from 0.5% to 1.3%, which is statistically superior to the 75% mortality rate of emergently managed splenic artery aneurysms in pregnant women. Stable aneurysms in the proximal third of the splenic artery are treated with simple ligation, with strong collateral blood supply negating the need for arterial reconstruction. Stable aneurysms in the middle third of the splenic artery can be managed with proximal and distal ligation, with the short gastric arteries usually providing enough circulation to preserve the spleen. Stable aneurysms in the distal third of the splenic artery require resection and splenectomy and possible pancreatectomy [1]. Ruptured splenic artery aneurysms are first treated with resuscitative measures followed by laparotomy, resection of aneurysm, and splenectomy [1]. If surgical management is delayed, crystalloid and blood transfusion products can be used in resuscitation efforts [2]. Neonatal support should be considered in pregnant patients, and critical care transport to a referral center with appropriate neonatal intensive care unit services should be arranged.

## Figures and Tables

**Figure 1:**